# Plants adapted to warmer climate do not outperform regional plants during a natural heat wave

**DOI:** 10.1002/ece3.2183

**Published:** 2016-05-23

**Authors:** Anna Bucharova, Walter Durka, Julia‐Maria Hermann, Norbert Hölzel, Stefan Michalski, Johannes Kollmann, Oliver Bossdorf

**Affiliations:** ^1^ Plant Evolutionary EcologyInstitute of Evolution & EcologyUniversity of TübingenTübingenGermany; ^2^ Department of Community EcologyHelmholtz Centre for Environmental Research‐UFZHalleGermany; ^3^ German Centre for Integrative Biodiversity Research (iDiv) Halle‐Jena‐LeipzigLeipzigGermany; ^4^ Restoration EcologyDepartment of Ecology & Ecosystem ManagementTechnische Universität MünchenMünchenGermany; ^5^ Biodiversity and Ecosystem Research GroupInstitute of Landscape EcologyUniversity of MünsterMünsterGermany

**Keywords:** Adaptation to novel environment, assisted migration, climate warming, global change, local adaptation, predictive provenancing

## Abstract

With ongoing climate change, many plant species may not be able to adapt rapidly enough, and some conservation experts are therefore considering to translocate warm‐adapted ecotypes to mitigate effects of climate warming. Although this strategy, called assisted migration, is intuitively plausible, most of the support comes from models, whereas experimental evidence is so far scarce. Here we present data on multiple ecotypes of six grassland species, which we grew in four common gardens in Germany during a natural heat wave, with temperatures 1.4–2.0°C higher than the long‐term means. In each garden we compared the performance of regional ecotypes with plants from a locality with long‐term summer temperatures similar to what the plants experienced during the summer heat wave. We found no difference in performance between regional and warm‐adapted plants in four of the six species. In two species, regional ecotypes even outperformed warm‐adapted plants, despite elevated temperatures, which suggests that translocating warm‐adapted ecotypes may not only lack the desired effect of increased performance but may even have negative consequences. Even if adaptation to climate plays a role, other factors involved in local adaptation, such as biotic interactions, may override it. Based on our results, we cannot advocate assisted migration as a universal tool to enhance the performance of local plant populations and communities during climate change.

## Introduction

Climate change is posing serious threats to biodiversity (Parmesan 2006), and many species may not be able to adapt rapidly enough to the changing environmental conditions (Hulme 2005). If species cannot evolve fast enough, they will have to migrate in order to survive. To avoid species extinctions, some researchers and conservation managers are advocating the intentional translocation of species or populations, a concept termed assisted migration (Hewitt et al. 2011). Assisted migration may either involve the movement of species outside their current range, or the translocation of populations or genotypes within a species' range (Gray et al. 2011; Pedlar et al. 2012; Williams and Dumroese 2013). The latter is the focus of our paper.

The strategy of assisted migration is based on the assumption that organisms are primarily adapted to their local climates. If the climate changes, local populations will become maladapted, and ecotypes from other locations with a climate similar to the novel conditions in the target locality are expected to perform better than the local populations. In order to maintain ecosystem productivity and functioning, those better adapted ecotypes from other locations should thus be intentionally introduced into the target populations (Sgrò et al. 2011).

In plant population and community restoration, the most common current practice is the use of local seed sources. The strategy is based on the large body of evidence for local or regional adaptation in plants (reviewed in Leimu and Fischer 2008; Hereford 2009). However, with ongoing climate change, the idea of replacing local plant sources with such from more warm‐adapted origins, is receiving increasing attention, and the potential risks and benefits of this approach have been the subject of intense debate (Kreyling et al. 2011; Frascaria‐Lacoste and Fernández‐Manjarrés 2012). More and more researchers consider assisted migration a promising strategy and a suitable tool for adapting to climate change (McLachlan et al. 2007; Vitt et al. 2009; Gray et al. 2011; Aitken and Whitlock 2013; Gallagher et al. 2015). Practitioners are slowly catching up and have begun to develop policy frameworks for implementing assisted migration into management (Burbidge et al. 2011; Frascaria‐Lacoste and Fernández‐Manjarrés 2012; Williams and Dumroese 2013).

So far, most of the support for assisted migration comes from climate envelope models (Gray and Hamann 2011, 2012) or from models connecting experimental data on plant performance with climate (Wang et al. 2010; Iverson and McKenzie 2013; Ikeda et al. 2014; Chakraborty et al. 2015; Koralewski et al. 2015; Yang et al. 2015). However, the ultimate test of the effectivity of assisted migration is of course when plants adapted to the climate of another region outperform the local ones under climate change in a transplant experiment. Such experiments are difficult to perform because they require either experimental climate manipulation, or the data have to be gathered under natural and necessarily unpredictable extreme weather events. Hence, such data are scarce, and so far the results are contradictory. While some studies confirmed a better performance of warm‐adapted genotypes (Schreiber et al. 2013; Lu et al. 2014; Wilczek et al. 2014), others either found no differences, or even better performance of the local plants (Beierkuhnlein et al. 2011; Hancock and Hughes 2014).

Here, we present data from a study in which we compared the performance of different ecotypes of six common grassland species under an unusual summer heat wave in Central Europe. Our study was part of a larger transplant experiment, with multiple ecotypes of multiple species planted in four experimental sites across Germany (Bucharova et al. [Link]). Coincidentally, 2013, the year of the main experiment, was one of the warmest years on record, with summer temperatures 1.4–2.0°C above the long‐term means in all experimental sites. This provided us with a unique opportunity to compare the performance of regional versus warm‐adapted ecotypes for multiple plant species in multiple locations and thus to test the hypothesis that under future climatic conditions, plants from warmer origins will outperform local plants.

## Methods

### Seed material

In Germany, a regional seed provenancing approach for grassland restoration has recently been established, where the country is divided into eight regions, within which seed transfers for grassland restoration takes place (for details see Bucharova et al. [Link]; Durka et al. [Link]). Native seeds of many common grassland species are now commercially available for each region. For our study, we chose seven common perennial grassland species frequently used in restoration: *Arrhenatherum elatius* (L.) P.B. ex J. et C. Presl, *Centaurea jacea* L., *Daucus carota* L.*, Galium album* Mill., *Hypochaeris radicata* L., *Knautia arvensis* (L.) Coult., and *Lychnis flos‐cuculi* (L.) Greuter and Burdet. All species are typical perennial plants of the temperate zone, with a growing season from spring to autumn (April to October) and peak growth and flowering in the summer. For each species, we obtained seeds from all (or most of) the eight geographic regions. The seeds were purchased from a certified regional seed producer (Rieger‐Hoffmann GmbH, Blaufelden, Germany).

### Common garden experiment

In the summer of 2013, we carried out parallel common garden experiments in four sites in Germany, each located in a different region. These experimental sites were 200–550 km apart from each other and differed significantly in climate. In each site, we germinated seeds in a greenhouse and then transplanted 12 seedlings per species and origin into pots filled with a standard potting soil (same pots and soil used in all sites). At the end of May 2013, we placed the young plants outside in a fully randomized block design. To avoid drought‐related mortality, the pots were watered when needed during the hottest summer period. Some plants died during the experiment, but overall mortality was low (<5%), and similar across origins. In early September 2013, toward the end of the growing season, when most of the plants started to wither, we harvested all plants, counted the number of inflorescences (individual flowers for *Lychnis*) per individual, cut the aboveground biomass, dried it at 70°C for 48 h, and weighed it. We used both biomass and the number of inflorescences as measures of overall plant performance and fitness. For number of inflorescences, we included only flowering plants, because we were working with perennial plants and only over one growing season. Some plants might simply not flower yet in the first year but accumulate resources (biomass) for flowering in the next year. Assessing performance as number of inflorescences is thus meaningful only in plants that do flower, whereas a lack of flowering does not mean low performance. On the other hand, biomass describes fitness rather well in plants that mostly invest resources into vegetative growth instead of flowering. For further details, see Bucharova et al. ([Link]).

### Defining regional and warm‐adapted ecotypes

The experiment was originally established to test for genetic differentiation and general regional adaptation, and these more general results have been published elsewhere (Bucharova et al. [Link]), together with a sister paper on molecular genetic differentiation (Durka et al. [Link]). However, the year 2013, in which we conducted our study, was one of the warmest years on record (http://www.ncdc.noaa.gov/sotc/global/2013/13), with summer (June–August) temperatures in our experimental sites 1.4–2.0°C higher than the respective long‐term means. To characterize the long‐term temperature conditions in each experimental and seed collection site, we used the 50‐year averages of summer (June–August) temperatures provided by the WorldClim database (Hijmans et al. 2005). To characterize the 2013 summer weather in each experimental site, we used data from the nearest meteorological station (www.dwd.de).

In each experimental site, we defined the ecotypes originating from the same region as where the site was located as the regional ecotypes for that site. In order to directly compare regional with warm‐adapted ecotypes, we selected one corresponding warm‐adapted ecotype of the seven available nonregional ecotypes. Our rationale for selecting the warm‐adapted ecotype was that it should originate from a collection site with a long‐term summer temperature at least 1°C above the long‐term temperature of the respective garden. If several ecotypes met this requirement, we selected the ecotype in which the long‐term temperature of origin was closest to the 2013 temperature in the experimental site. If no ecotype met this requirement, the specific site was not included in the analysis for that species. Following this procedure, we selected the ecotypes from region 6 (Upper Rhine Basin) as warm‐adapted ecotypes for all species in all sites (Table 1). The geographic distances between the origins of the warm‐adapted ecotypes and the locations of the corresponding common gardens were between 100 and 450 km. As *Knautia arvensis* was represented by only one garden in the final dataset, we excluded this species from our analyses.

**Table 1:** The summer temperature anomalies (2013 June–August temperature minus long‐term June–August mean) in each site, as well as the differences between the long‐term temperature means of each site and the seed origins of warm‐adapted ecotypes. Missing values indicate that no warm‐adapted ecotype (≥1°C above long‐term mean) could be identified

	Freising	Halle	Münster	Tübingen
Temperature anomaly 2013	+1.4°C	+1.6°C	+1.8°C	+2.0°C
*Arrhenaterum elatius*	+1.4	–	+1.6	+1.3
*Centaurea jacea*	+2.0	+1.2	+2.0	+1.9
*Daucus carota*	+2.1	+1.3	+2.3	+2.0
*Galium album*	–	+1.3	+2.3	+2.0
*Hypochaeris radicata*	+1.5	–	+1.7	+1.4
*Lychnis flos‐cuculi*	+2.2	–	+2.4	+2.1

### Data analysis

We analyzed the biomass and inflorescences data of each species through generalized linear mixed models, using the *lme4* package in R (R Development Core Team 2009), that included ecotype (regional vs. warm‐adapted) as fixed factor, and block, garden and ecotype identity as random factors. We used models with normal and Poisson error distribution, respectively. To obtain a quantitative measure of the relative advantage of warm‐adapted ecotypes over the regional ones, we used the effect sizes (back‐transformed in the case of the inflorescences data), divided by the average value for regional ecotypes. The resulting values thus expressed the percentage change from regional to warm‐adapted ecotypes. We calculated credible intervals, a Bayesian analogue of confidence intervals, for these values based on 10,000 simulations of the mean and variance of each estimate, using the *sim* function in the R package *arm* (for details see Korner‐Nievergelt et al. 2015). If these credible intervals did not include 0, we considered the difference between the two ecotypes to be significant. The approach is more reliable for testing significance of fixed factors in GLMM than the *P*‐values from classical likelihood ratio tests (Bolker et al. 2009).

## Results

**Figure 1:**
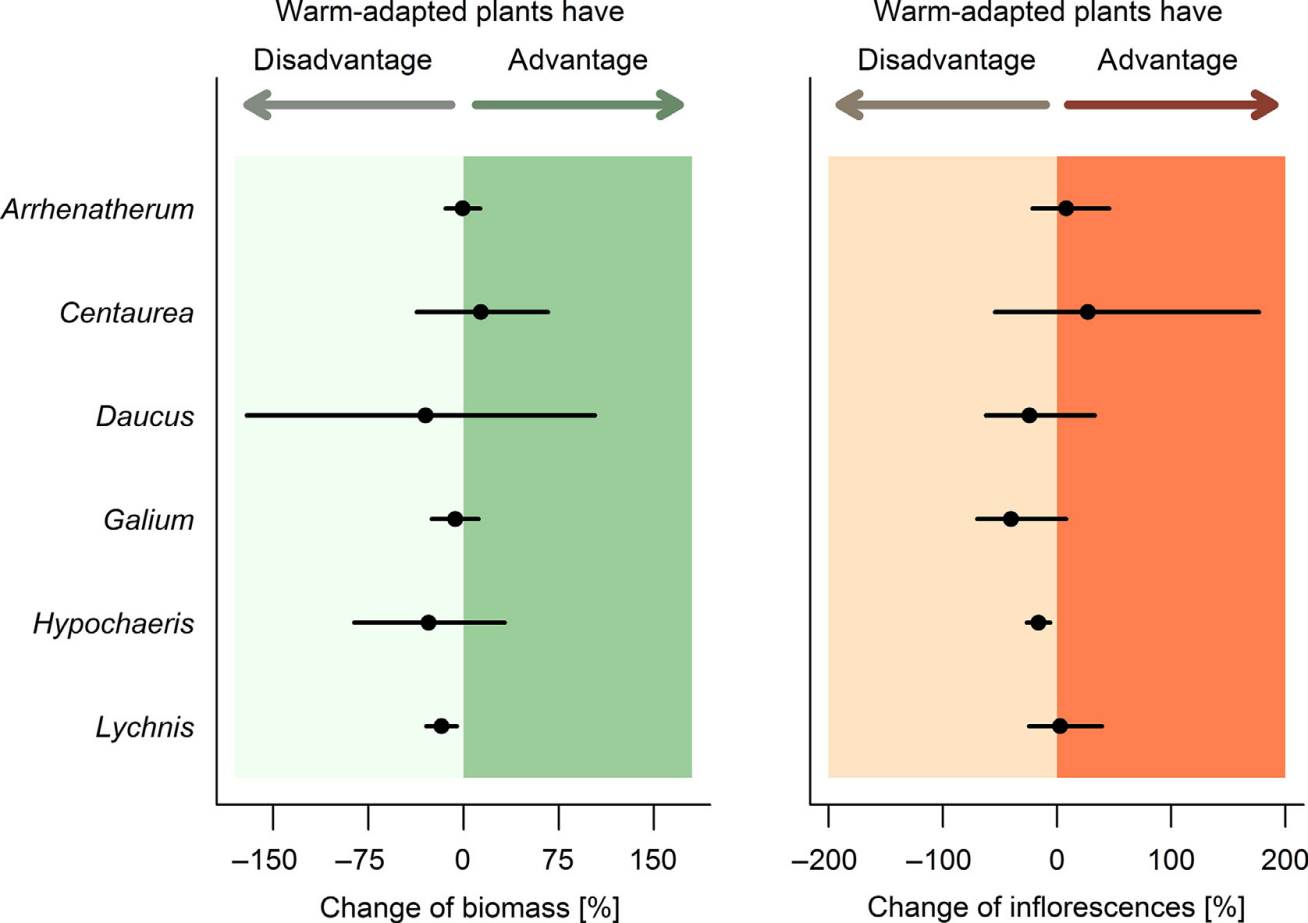
Differences in performance (biomass and number of inflorescences) between warm‐adapted and regional ecotypes. The values are based on effect sizes obtained from GLMM. The error bars are Bayesian credible intervals which indicate significance if they exclude 0.

In four of the six studied species, there was no significant difference in performance, measured by total aboveground biomass or the number of inflorescences produced, between warm‐adapted and regional ecotypes (Fig. 1). In the two remaining cases, the warm‐adapted ecotypes showed significantly lower performance than the regional ecotypes: −17.6% biomass in *Lychnis* and −16.4% inflorescences in *Hypochaeris*. Among the nonsignificant effects, there were also more pointing toward a disadvantage of warm‐adapted plants than toward the opposite (Fig. 1).

## Discussion

To test the potential benefit of translocating plant ecotypes in response to climate warming, we compared the performance of warm‐adapted, nonregional ecotypes of six common grassland species to the performance of regional ecotypes of the same species in an exceptionally warm year. If adaptation to climate is one of the main determinants of local plant performance, then warm‐adapted plants should exhibit higher fitness than regionally adapted plants under elevated temperature conditions. However, our results did not confirm this hypothesis. Regional plants – which were expected to be long‐term adapted to 1–2°C less than the temperatures experienced during our experiment – performed either equally well or even outperformed the warm‐adapted plants. This indicates that even in a warmer climate, the regional plants benefit from other, climate‐unrelated aspects of regional adaptation. Similar results have been reported previously for five perennial grasses (Beierkuhnlein et al. 2011; Hancock and Hughes 2014). On the other hand, warm‐adapted ecotypes have been shown to have an advantage in two tree species (Schreiber et al. 2013; Lu et al. 2014) as well as in *Arabidopsis thaliana*, a short‐lived annual with known adaptation to climate (Wilczek et al. 2014). It is possible that there are fundamental differences between growth forms and life histories, and our data, together with that of Beierkuhnlein et al. (2011) and Hancock and Hughes (2014), indicate that it might be particularly herbaceous perennials for which assisted migration might not suitable. However, although our study significantly increased the total number of species for which an assisted migration of warm‐adapted ecotypes has now been tested, we still need more data before we can conclude that these are truly general patterns that distinguish taxonomic or functional groups.

Although our results are in good agreement with several previous studies, some caution is necessary. First, we carried out the experiment over only one growing season, which means that we may not have seen the whole picture, and the longer‐term responses of different ecotypes could have differed from those observed in the first year, particularly if plants would only have flowered in the second year. Still, the majority of the plants in our study flowered and set fruit, and thus, the elevated temperature affected most of their life cycle. Second, in each species, we used the same warm‐adapted ecotype in all four gardens. While this approach is somewhat similar to previous studies that compared a single local ecotype with multiple foreign ones (e.g., Beierkuhnlein et al. 2011; Weißhuhn et al. 2012; Hancock et al. 2013; Hancock and Hughes 2014), it raises the question whether this single warm‐adapted ecotype was not especially poor *per se*. However, we worked with six species, and it appears very unlikely that this can explain a consistent nonadvantage across multiple species. Moreover, our data from the larger experiment (Bucharova et al. [Link]) show that this was not the case, as ecotypes from this region did not perform consistently worse compared with other genotypes, except in *Daucus*. To further confirm this, we redid the same analyses as above, but this time we defined as warm‐adapted ecotypes those that came from any origin that was warmer than the origin of the regional plants. The results are generally consistent with our previous findings (Fig. S1), with no overall advantage, or sometimes even disadvantages of warm‐adapted ecotypes.

In two of our studied species, *Hypochaeris* and *Lychnis*, the regional plants outperformed warm‐adapted, nonregional plants and thus showed evidence for regional adaptation, even during the extreme summer heat wave of 2013. As water and soil conditions were under our experimental control, there are only few environmental factors left that can be behind the observed regional adaptation, for example, altitude, light conditions (e.g., different amounts of sunshine hours or their temporal patterns), or biotic interactions. We think that the best candidate here is biotic interactions. Adaptation to herbivores and pathogens is known to be an important part of the local adaptation of many plant species (Laine 2008; Garrido et al. 2012; Grassein et al. 2014).

The strategy of assisted migration is based on the assumption that climate adaptation is the key driver of plant performance (Hewitt et al. 2011; Sgrò et al. 2011). Consequently, most practical recommendations rely on climatic models (Wang et al. 2010; Iverson and McKenzie 2013; Ikeda et al. 2014; Chakraborty et al. 2015; Koralewski et al. 2015; Yang et al. 2015). Our results show that this can be misleading and that the translocation of warm‐adapted ecotypes will not necessarily lead to an increase in performance in a warmer climate but that performance might even decrease, presumably because other adaptations to local or regional environmental conditions or biotic interactions play an even greater role than climate adaptation for the performance of the plants.

In line with several previous studies, our results show that the possible benefits of assisted migration are species‐dependent to say the least. To evaluate the general value of assisted migration as a management tool, we need more large‐scale experiments comparing the performance of warm‐adapted and regional ecotypes under climate change conditions across many species and life‐history types. Only large‐scale multispecies studies will provide the necessary evidence to derive general guidelines and recommendations for climate change management. However, so far such data are not available, and the only means of evaluating the prospects of success of a planned assisted migration are field experiments, which ideally should be carried out prior to any assisted migration. In the absence of further evidence, we suggest that the use of regionally sourced seeds may still be the best default solution in order to avoid doing more harm than good.

## Supporting information


**Table S1**. Average biomasses and numbers of inflorescences (±SE) of all studied ecotypes.
**Figure S1**. Differences in performance (biomass and number of inflorescences) between warm‐adapted and regional ecotypes, using all plants from warmer origins as warm‐adapted ecotypes.
