# Supplementary material for: Plants adapted to warmer climate do not outperform regional plants during a natural heat wave
Source: Ecol Evol. 2016 May 23;6(12):4160–5. doi: 10.1002/ece3.2183 (PMC4880551; doi:10.1002/ece3.2183)

**Table S1:** Average biomasses and numbers of inflorescences (+ S.E.) of all studied ecotypes. The numbers in the table represent the average performances of each of the five ecotypes across all four experimental gardens and thus represent overall performances of the different ecotypes.

|  | | ARR | | | CEN | | | DAU | | | GAL | | | HYP | | | LYC | | |
| --- | --- | --- | --- | --- | --- | --- | --- | --- | --- | --- | --- | --- | --- | --- | --- | --- | --- | --- | --- |
| *Biomass [g]* | | | |  | | |  | | |  | | |  | | |  | | |  |
| Regional ecotypes | Freising | | 17.3+1.5 | | | 16.8+1.8 | | | 26.1+3.6 | | | - | | | 18.9+1.9 | | | 14.3+1.0 | |
|  | Halle | | 19.1+1.5 | | | 24.6+3.5 | | | 33.7+4.7 | | | 13.9+0.9 | | | 17.0+1.5 | | | - | |
|  | Münster | | 18.5+1.7 | | | 22.9+3.5 | | | 25.0+3.4 | | | 10.1+0.6 | | | 18.2+1.6 | | | 16.9+1.1 | |
|  | Tübingen | | 20.0+1.6 | | | 16.5+2.1 | | | 25.7+4.3 | | | 14.2+1.2 | | | 21.3+1.9 | | | 16.8+1.3 | |
| Warm-adapted ecotypes | | | 18.1+1.4 | | | 27.0+3.3 | | | 19.5+2.9 | | | 16.0+1.3 | | | 18.3+2.1 | | | 15.2+0.9 | |
| *# Inflorescences* | | |  | | |  | | |  | | |  | | |  | | |  | |
| Regional ecotypes | Freising | | 13.74+1.4 | | | 40.34+4.8 | | | 31.04+3.4 | | | - | | | 60.83+5.6 | | | 169.29+12.1 | |
|  | Halle | | 15.53+1.6 | | | 34.67+5.1 | | | 35.47+3.7 | | | 6.07+1.4 | | | 76.51+5.7 | | | - | |
|  | Münster | | 14.17+1.3 | | | 43.12+6.7 | | | 25.15+3.8 | | | 2.25+0.36 | | | 68.38+6.6 | | | 143.28+12.1 | |
|  | Tübingen | | 15.38+1.7 | | | 26.78+3.5 | | | 33.21+3.4 | | | 13.84+1.8 | | | 63.04+4.9 | | | 145.44+19.1 | |
| Warm-adapted ecotypess | | | 15.23+1.4 | | | 58.61+7.4 | | | 23.82+3.0 | | | 4.09+0.7 | | | 61.98+5.9 | | | 206.78+16.3 | |

Figure S1: Differences in performance (biomass and number of inflorescences) between warm-adapted and regional ecotypes, using all plants from warmer origins as warm-adapted ecotypes. The values are based on effect sizes obtained from GLMM. The error bars are Bayesian credible intervals which indicate significance if they exclude 0. In most cases (7 out of 12) warm-adapted plants performed similar to regional ones, but in four cases they performed significantly worse. Only in one case (inflorescenses of *Daucus*) warm-adapted plants outperformed regional ones.


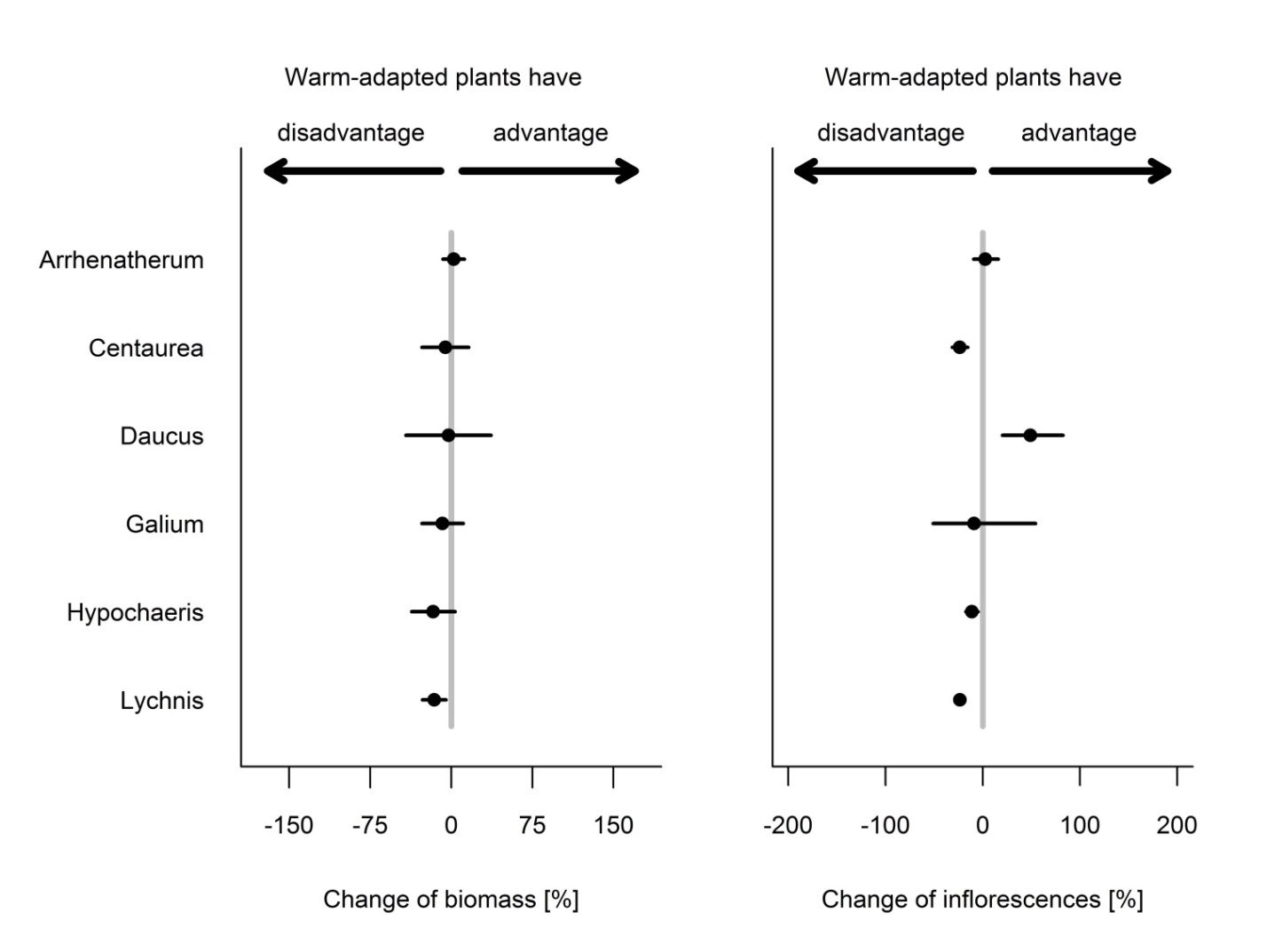

Supplement: Supplementary file 1 — Table S1. Average biomasses and numbers of inflorescences (±SE) of all studied ecotypes. Figure S1. Differences in performance (biomass and number of inflorescences) between warm‐adapted and regional ecotypes, using all plants from warmer origins as warm‐adapted ecotypes. [file ECE3-6-4160-s001.docx]
